# FTO alleviated the diabetic nephropathy progression by regulating the N6-methyladenosine levels of DACT1

**DOI:** 10.1515/biol-2022-1049

**Published:** 2025-05-05

**Authors:** Xuanwen Li, Qing Huang, Shinong Gu, Ping Zheng

**Affiliations:** Department of Nutrition, Tianjin Beichen Traditional Chinese Medicine Hospital, Tianjin, China; College of Environment and Public Health, Xiamen Huaxia University, Xiamen, Fujian, China; Department of Nutrition, Tianjin Third Central Hospital, No. 83, Jintang Road, Hedong District, Tianjin, 300170, China

**Keywords:** diabetic nephropathy, FTO, DACT1, IGF2BP1

## Abstract

Diabetic nephropathy (DN) is one of the most important microvascular complications of diabetes. The role of epigenetic regulation in DN has attracted much attention recently. This research was performed to explore the role of FTO in the DN progression. The renal tissues of DN patients were collected and the podocytes were stimulated with high glucose (HG) to establish the DN model *in vitro*. Western blot along with reverse transcription quantitative polymerase chain reaction assays was performed to analyze the mRNA as well as protein expressions. Immunohistochemistry and immunofluorescence were carried out to measure the FTO and DACT1 levels. The interaction between FTO/IGF2BP1 and DACT1 was verified by double luciferase reports and RNA-binding protein immunoprecipitation assays. FTO was declined, and DACT1 was enhanced in the HG-treated podocytes as well as renal tissues of DN patients. Overexpressed FTO declined the mRNA levels of MCP-1, IL-6, TNF-α, and the apoptosis rate of HG-treated podocytes. The N6-methyladenosine (m6A) levels, mRNA expression, and stability of FTO were depleted after FTO overexpression. DACT1 overexpression reversed the function of oe-FTO in podocytes stimulated with HG. Furthermore, IGF2BP1 knockdown declined the mRNA expression as well as the stability of FTO. In conclusion, FTO-medicated m6A modification of DACT1 was dependent on IGF2BP1 in DN progression.

## Graphical abstract

**Figure:**
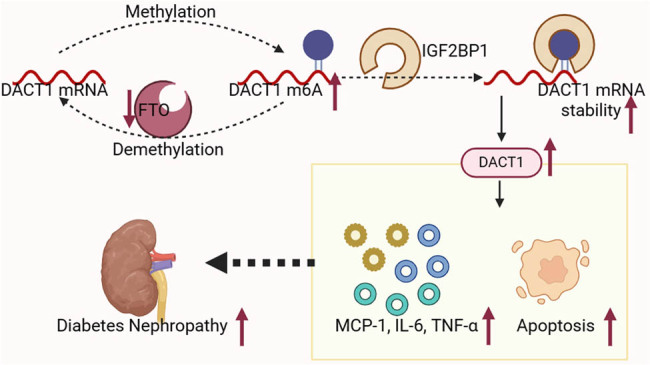

## 1 Introduction

Diabetic nephropathy (DN) is a leading microvascular complication of diabetes mellitus, characterized by chronic kidney disease that can progress to end-stage renal disease (renal failure) [1]. In addition, DN causes a variety of cardiovascular diseases, which greatly affect the survival and prognosis of patients [2]. The primary renal manifestations of DN include glomerular dysfunction, glomerulosclerosis, and impaired glomerular filtration [3]. Emerging evidence suggests that abnormalities in podocyte function and structure serve as reliable indicators of DN progression, offering potential biomarkers for early detection and intervention [4]. Podocytes, also known as visceral epithelial cells of the renal capsule, are critical components of the glomerular filtration barrier [5,6]. They play an essential role in maintaining the integrity of this barrier and regulating glomerular filtration. Damage to podocytes and mesangial cells compromises the filtration barrier, leading to dysregulated glomerular filtration and exacerbating the progression of DN [7]. Clinical studies have confirmed that podocyte injury results in persistent proteinuria and a decline in the glomerular filtration rate in patients with DN [8]. Consequently, targeting podocyte injury represents a promising therapeutic strategy to mitigate DN progression.

The role of epigenetic regulation in DN has garnered significant attention in recent years [9]. Epigenetic modifications, which include DNA methylation, RNA modifications, and protein modifications, operate at both the genomic and transcriptomic levels to influence post-transcriptional gene regulation [10]. Among the 160 known RNA modifications, N6-methyladenosine (m6A) is the most prevalent *in vivo* [11]. The biological effects of m6A modification mainly involve m6A methylase, m6A demethylase, and methylated reading protein [12]. One key player in m6A demethylation is the fat mass and obesity-associated protein (FTO), which primarily localizes to the cell nucleus [13]. FTO has been implicated in the development of diabetes-related diseases [14]. In patients with diabetes, a high-fat diet increases FTO expression, leading to reduced m6A methylation levels. This promotes the expression of genes involved in lipid metabolism, thereby elevating serum glucose levels and exacerbating the pathological progression of diabetes [15]. However, the potential mechanism of FTO in DN remains unclear.

Dapper homolog 1 (DACT1), located on chromosome 14q23 1 region, is a recently identified tumor suppressor gene that exhibits downregulation in various types of malignant tumors [16–18]. The protein encoded by DACT1 plays a critical role in regulating cell proliferation, differentiation, and apoptosis [19]. Beyond its involvement in cancer, DACT1 has been implicated in other diseases, including human muscle disorders [20] and type 2 diabetes mellitus [21]. Recent studies have highlighted the importance of epigenetic modifications, particularly m6A methylation, in the regulation of gene expression. Lv et al. [22] demonstrated that FTO decreased the mRNA expression and stability of DACT1 by regulating the m6A methylation levels in osteosarcoma. Therefore, we speculated that FTO may also play the same regulatory role on DACT1 in DN. This research was carried out to analyze the specific mechanism of FTO in DN patients and high glucose (HG)-treated podocytes.

## 2 Materials and methods

### 2.1 Tissues collection

Renal tissues from ten DN patients were collected at Tianjin Beichen Traditional Chinese Medicine Hospital. Additionally, the ten renal tissue samples were obtained from the healthy portions of nephrectomy specimens removed during surgery for renal cell carcinoma. Collected tissues were immediately snap-frozen in liquid nitrogen and stored at −80°C for subsequent experiments.


**Informed consent:** Informed consent has been obtained from all individuals included in this study.
**Ethical approval:** The research related to human use has been complied with all the relevant national regulations and institutional policies and in accordance with the tenets of the Helsinki Declaration and has been approved by the Ethics Committee of Tianjin Beichen Traditional Chinese Medicine Hospital.

### 2.2 Immunohistochemistry staining

Renal tissues embedded in paraffin were cut into 4-μm-thick slices and then deparaffinized and rehydrated through graded alcohols. The slices were then washed twice in phosphate-buffered saline (PBS) for 10 min. After that, the slices were treated with primary antibodies against FTO (Abcam, Cambridge, MA, USA) and DACT1 (Abcam). After washing, the slices were further incubated with the anti-mouse IgG H&L (HRP) secondary antibody (Abcam) at 37°C for 30 min. For visualization, the slices were stained with 3,3′-diaminobenzidine working solution for 3 min and then counterstained with hematoxylin. Finally, the slices were dehydrated, cleared, and observed under a microscope.

### 2.3 Cell culture and treatment

Conditionally immortalized human podocytes were kindly provided by Professor Min Jiang of Southeast University. The podocyte cells were maintained in RPMI 1640 (Invitrogen, CA, USA) supplemented with 10% FBS (37°C and 5% CO_2_). For DN model establishment, the podocyte cells were stimulated with 35 mmol/L HG for 12 h.

The FTO overexpression vector (oe-FTO), DACT1 overexpression vector (oe-DACT1), and short hairpin RNAs (sh-IGF2BP1, IGF2BP2, IGF2BP3, YTHDF1, YTHDF2, and YTHDC1), and negative controls (oe-NC and sh-NC) were purchased from Genscript (Nanjing, China). Lipofectin3000 (Invitrogen) was applied for the promotion of the transfection efficacy of these cells.

### 2.4 qRT-PCR

RNAs were obtained using TRIzol (Beyotime, Shanghai, China). Then, the reverse transcription was performed according to the instructions of the RT Master Mix for quantitative real-time PCR (qPCR) II (MedChemExpress, Monmouth Junction, NJ, USA). SYBR Green qPCR Master Mix (MedChemExpress) was utilized to measure the target gene expressions on a CFX384 real-time polymerase chain reaction (PCR) system (Bio-Rad, Hercules, CA, USA). GAPDH was selected as the control, and the primer sequence is shown as follows: METTL3, forward primer: 5′-TTGTCTCCAACCTTCCGTAGT-3′, reverse primer: 5′-CCAGATCAGAGAGGTGGTGTAG-3′; WTAP, forward primer: 5′-CTTCCCAAGAAGGTTCGATTGA-3′, reverse primer: 5′-TCAGACTCTCTTAGGCCAGTTAC-3′; METTL14, forward primer: 5′-GAACACAGAGCTTAAATCCCCA-3′, reverse primer: 5′-TGTCAGCTAAACCTACATCCCTG-3′; FTO, forward primer: 5′-ACTTGGCTCCCTTATCTGACC-3′, reverse primer: 5′-TGTGCAGTGTGAGAAAGGCTT-3′; DACT1, forward primer: 5′-TTGAACTGTTTGAGGCGAAGAG-3′, reverse primer: 5′-ACTGAACACCGAGTTAGAGGAAT-3′; MCP-1, forward primer: 5′-CAGCCAGATGCAATCAATGCC-3′, reverse primer: 5′-TGGAATCCTGAACCCACTTCT-3′; IL-6, forward primer: 5′-AGGAAGGGCCGTCTATCAATC-3′, reverse primer: 5′-CACTGTCACTTCGTGGAACTG-3′; TNF-α, forward primer: 5′-CGGCTACCTAGTCTACGCC-3′, reverse primer: 5′-AAGTCGCCGCCAATGTTGA-3′. The Reaction procedure was as follows: predenaturation at 95°C for 30 s; PCR was carried out at 95°C for 15 s and 60°C for 30 s for 40 cycles. The 2^–ΔΔCT^ method was used to analyze the data. Furthermore, the cells were treated with actinomycin D for 2, 4, 6, 8 h. After that, the mRNA levels of DACT1 were detected with reverse transcription quantitative polymerase chain reaction (RT-qPCR), respectively.

### 2.5 Lactate dehydrogenase (LDH) release

The LDH release from cells was quantified using the LDH Cytotoxicity Assay Kit (Abcam, USA) according to the manufacturer’s instructions. The assay measures the activity of LDH released into the culture supernatant as an indicator of cell membrane integrity and cytotoxicity.

### 2.6 Cell viability determination

To assess the viability of podocytes, a Cell Counting Kit-8 (CCK-8) assay was employed. Podocytes were seeded in 96-well plates at a density of 5 × 10^3^ cells per well and allowed to adhere overnight. Following treatment with designated compounds or under experimental conditions, cells were incubated with 10 μL of CCK-8 solution (Dojindo Molecular Technologies, Japan) per 100 μL of culture medium for 2 h at 37°C in a humidified atmosphere containing 5% CO_2_. Absorbance was measured at 450 nm using a microplate reader (BioTek Instruments, USA). Untreated cells served as controls, and each condition was tested in triplicate. Viability was expressed as a percentage relative to control cells.

### 2.7 Terminal deoxynucleotide transferase‐mediated dUTP nick end labeling (TUNEL) assay

The apoptotic condition of podocytes was detected by using a TUNEL cell death detection kit (Roche, Basel, Switzerland). In brief, podocytes were fixed with paraformaldehyde, first permeating them with 0.1% Triton X‐100 for 2 min and then adding the TUNEL reaction mixture and incubating for 1 h to stain the apoptotic cells. All nuclei were stained with DAPI. The three random fields were observed in each sample under the microscope. Red fluorescence was observed at 620 nm and blue fluorescence was observed at 460 nm. Apoptosis index = number of cells stained by TUNEL/total number of cells.

### 2.8 Western blot

Total protein extraction was completed with RIPA buffer (Thermo Fisher Scientific), and the protein quantification was carried out obeying the protocols of the BCA assay (Pierce, USA). Equivalent samples (50 μg/well) were detached on 10% SDS–PAGE, followed by transferring to PVDF membranes (Millipore, USA), and blocked with 5% skim milk. Next, the membranes were treated with primary antibodies (METTL3, 1:1,000; WTAP, 1:500; METTL14, 1:800; FTO, 1:1,200; ALKBH5, 1:600; DACT1, 1:2,000; β-Actin, 1:4,000; Abcam, USA) on shaking table overnight at 4°C. After washing the membranes the next day, the membranes were incubated with the corresponding secondary antibody for 1 h. Finally, ECL luminescent solution (Beyotime) was added for reaction for 2 min. A Bio-Rad exposure instrument was used for chemiluminescence exposure. The results were analyzed by image lab program and β-actin was used as control.

### 2.9 Cell apoptosis determination

The density of podocytes was adjusted to 2.5 × 10^5^/mL, transferred into six-well plates, and cultured for 72 h. The cells were then washed and resuspended with a binding buffer. Next, cells were treated with 5 μL Annexin V-FITC along with 10 μL PI in the dark for 10 min, respectively. Finally, the apoptosis rate was analyzed by flow cytometry.

### 2.10 M6A methylation level determination

The m6A methylation content in the total RNA of the cells was measured using the M6A Quantification Kit (AmyJet, Wuhan, China). All operations shall be carried out in strict accordance with the requirements of the kit.

### 2.11 RNA methylation immunoprecipitation (MeRIP) assay

The Magna-MeRIP kit (A&D Technology Co., Ltd., Beijing, China) was used to detect the m6A methylation level of DACT1. Briefly, the cells were treated with magnetic beads and divided into the IgG group and the m6A group. Then, the cells were cultured with the corresponding anti-m6A. One hour later, the magnetic bead/antibody mixture was treated with cell lysate for 12 h at 4°C. Fragmented RNA was incubated with anti-m6A magnetic beads in an IP buffer at 4°C for 2 h. After washing, RNA was eluted and purified, and mRNA expression was measured using qPCR.

### 2.12 Luciferase reporter assay

The wild-type (WT) and mutant (MUT) sequences of DACT1 were inserted into the Pmir-GLO vector. The podocytes were co-transfected with WT or MUT vectors and oe-FTO or sh-IGF3BP1 using Lipofectamine 3000 for 48 h. In conformity with the manual of the Dual-Luciferase Reporter Assay Kit (Promega, USA), luciferase activity was examined after 48 h.

### 2.13 RNA-binding protein immunoprecipitation (RIP) assay

The relationship between FTO/IGF2BP1 and DACT1 was evaluated by a RIP kit (Geneseed, Guangzhou, China). Briefly, cells were lysed using a lysis buffer with protease inhibitor and RNase inhibitor for 10 min on ice. The supernatant was collected by centrifuging at 14,000 × *g* for 10 min. The supernatant (100 μL) was used as the Input group, and 900 μL supernatant was pre-treated with protein A + G beads at 4°C for 10 min. In addition, protein A + G beads (200 μL) were incubated with IgG or FTO/IGF2BP1 antibodies at 4°C for 2 h. The antibody-bead sample was incubated with the supernatant at 4°C for 12 h. After washing, RNA was extracted and the expression of DACT1 was measured by qPCR.

### 2.14 Immunofluorescence

When the cells in each group grew to 80%, the culture medium was discarded. Then, the cells were washed with PBS twice and fixed with 4% paraformaldehyde for 10 min. Next, the cells were permeated with 0.3% Triton-100 for 10 min. After blocking, the cells were incubated with primary antibody (DACT1) at 37°C for 1.5 h. After PBS rinsing, the cells were added with the corresponding secondary antibody and incubated at 37°C for 45 min. The cells were treated with DAPI and sealed with nail polish. The cells were observed and photographed under a fluorescence microscope.

### 2.15 Statistical analysis

The GraphPad Prism 8.0 software was used for data analysis. Each experiment was conducted independently three times. Data are shown as mean ± standard deviation. The significance was analyzed using an unpaired *t*-test between two groups and a one-way analysis of variance among multiple groups. *P* < 0.05 was statistical significance.

## 3 Results

### 3.1 FTO was increased in the HG-stimulated podocytes and renal tissues

**Figure 1:**
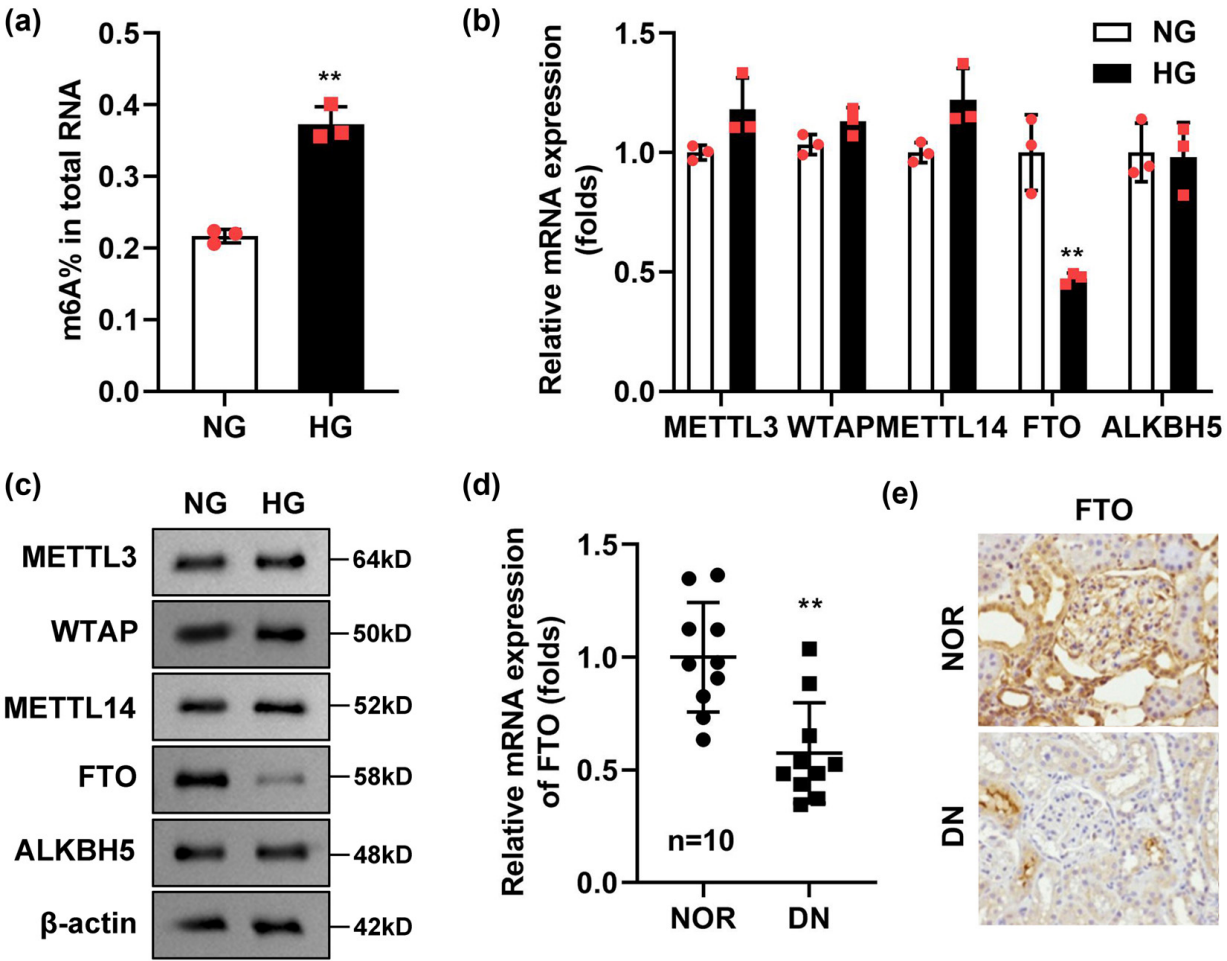
FTO was elevated in the HG-stimulated podocytes and renal tissues. (a) m6A methylation content was elevated in the HG-treated podocytes (*n* = 3). (b and c) The levels of m6A-related genes were determined with RT-qPCR and Western blot (*n* = 3). (d) FTO was elevated in the renal tissues of DN patients (*n* = 10). (e) The immunohistochemistry staining of FTO in the renal tissues of DN patients (*n* = 10). **P < 0.01.

To investigate the role of m6A methylation in DN, we established a DN model using podocytes stimulated with HG. Our results demonstrated that the total m6A methylation content was significantly increased in HG-treated podocytes compared to control cells (Figure 1a). Next, we analyzed the expression levels of genes involved in m6A methylation modification using RT-qPCR. We observed a marked reduction in FTO mRNA levels in HG-stimulated podocytes, while the expression levels of other m6A-related genes remained unchanged (Figure 1b). Consistent with these findings, Western blot analysis revealed a significant decrease in FTO protein levels in HG-treated podocytes (Figure 1c). To further validate these observations, we collected renal tissues from 10 DN patients and 10 healthy individuals whose tissues were removed during nephrectomy for renal cell carcinoma. Compared to healthy renal tissues, FTO expression was significantly reduced in the renal tissues of DN patients, as determined by both Western blot (Figure 1d) and immunohistochemistry (Figure 1e).

### 3.2 Overexpressed FTO alleviated the injury of HG-stimulated podocytes

**Figure 2:**
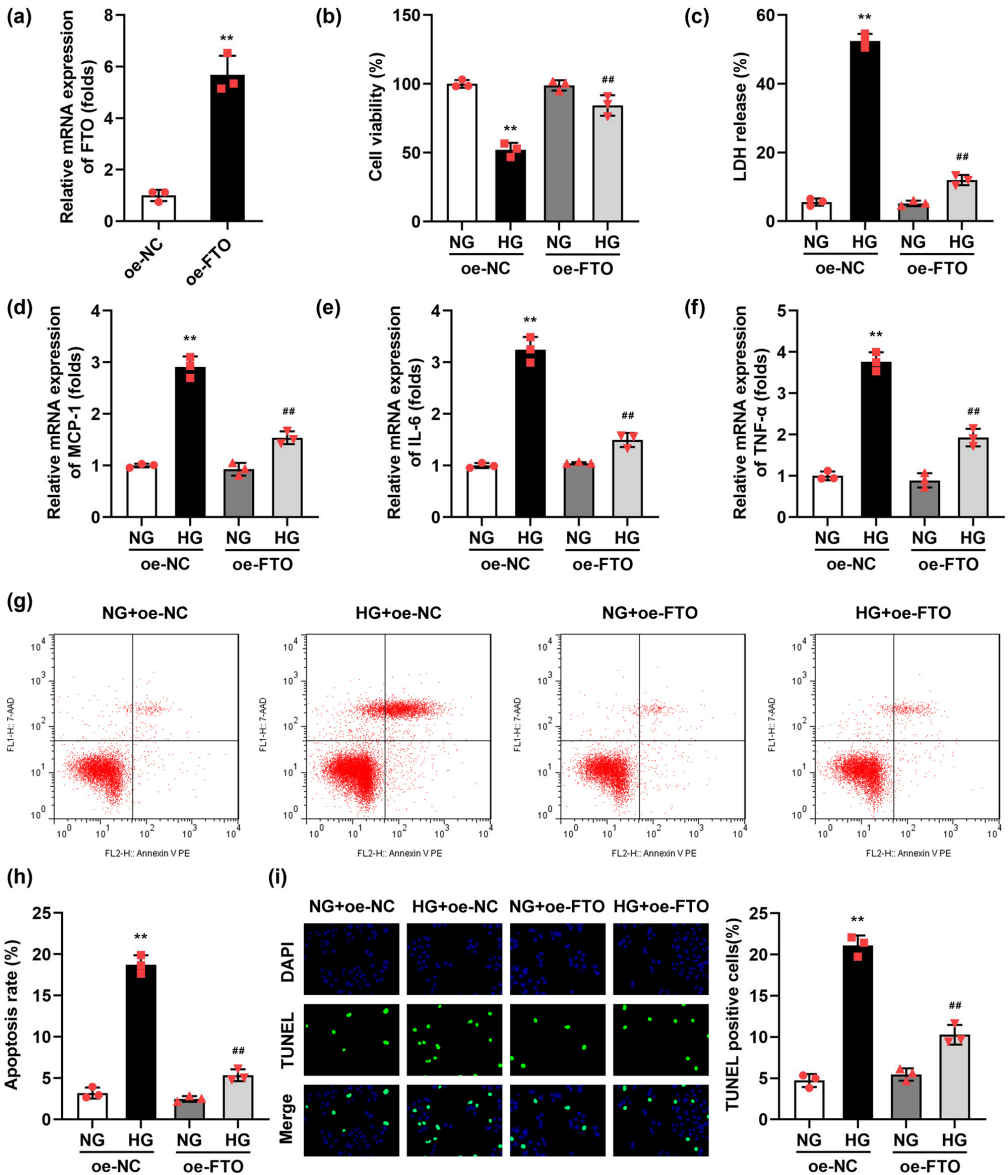
Overexpressed FTO relieved the injury of podocytes induced by HG. (a) Validation of transfection efficiency of oe-FTO. (b) The cell viability was detected by CCK-8 assay. (c) The LDH release was assessed with a kit. (d–f) The mRNA levels of MCP-1, IL-6, and TNF-α were tested with RT-qPCR. (g and h) Flow cytometry was performed to detect the cell apoptosis. (i) TUNEL staining was conducted to detect cell death (*n* = 3). **P < 0.01 vs NG + oe-NC group, ^##^ *P* < 0.01 vs HG + oe-FTO.

Given the significant reduction in FTO expression observed in DN, we hypothesized that enhancing FTO expression might alleviate the progression of DN. To test this hypothesis, we overexpressed FTO in a DN cell model to analyze its effects on cellular inflammatory responses and apoptosis levels. FTO was successfully overexpressed in podocytes, as evidenced by a substantial increase in FTO mRNA levels compared to control cells (Figure 2a). After HG stimulation, the LDH release (Figure 2b), mRNA expressions of MCP-1 (Figure 2c), IL-6 (Figure 2d), TNF-α (Figure 2e), apoptosis rate (Figure 2f and g), and TUNEL-positive cells (Figure 2h) were prominently elevated. The significant upregulation of MCP-1, IL-6, and TNF-α in the cells meant that HG has caused an inflammatory response in podocytes, which also explained the increase in apoptosis levels of podocytes may be caused by an inflammatory response. However, FTO overexpression prominently depleted the MCP-1 (Figure 2c), IL-6 (Figure 2d), TNF-α (Figure 2e) levels, and apoptosis rate (Figure 2f and g), as well as TUNEL-positive cells (Figure 2h). In addition, we obtained the podocytes from the renal tissues of DN patients and overexpressed FTO in the cells 2-fold. We found that FTO overexpression also significantly increased the cell viability and decreased the MCP-1, IL-6, TNF-α levels, apoptosis rate, and TUNEL-positive cells in the HG-treated podocytes (Figure S1).

### 3.3 DACT1 was elevated in the HG-stimulated podocytes and renal tissues

**Figure 3:**
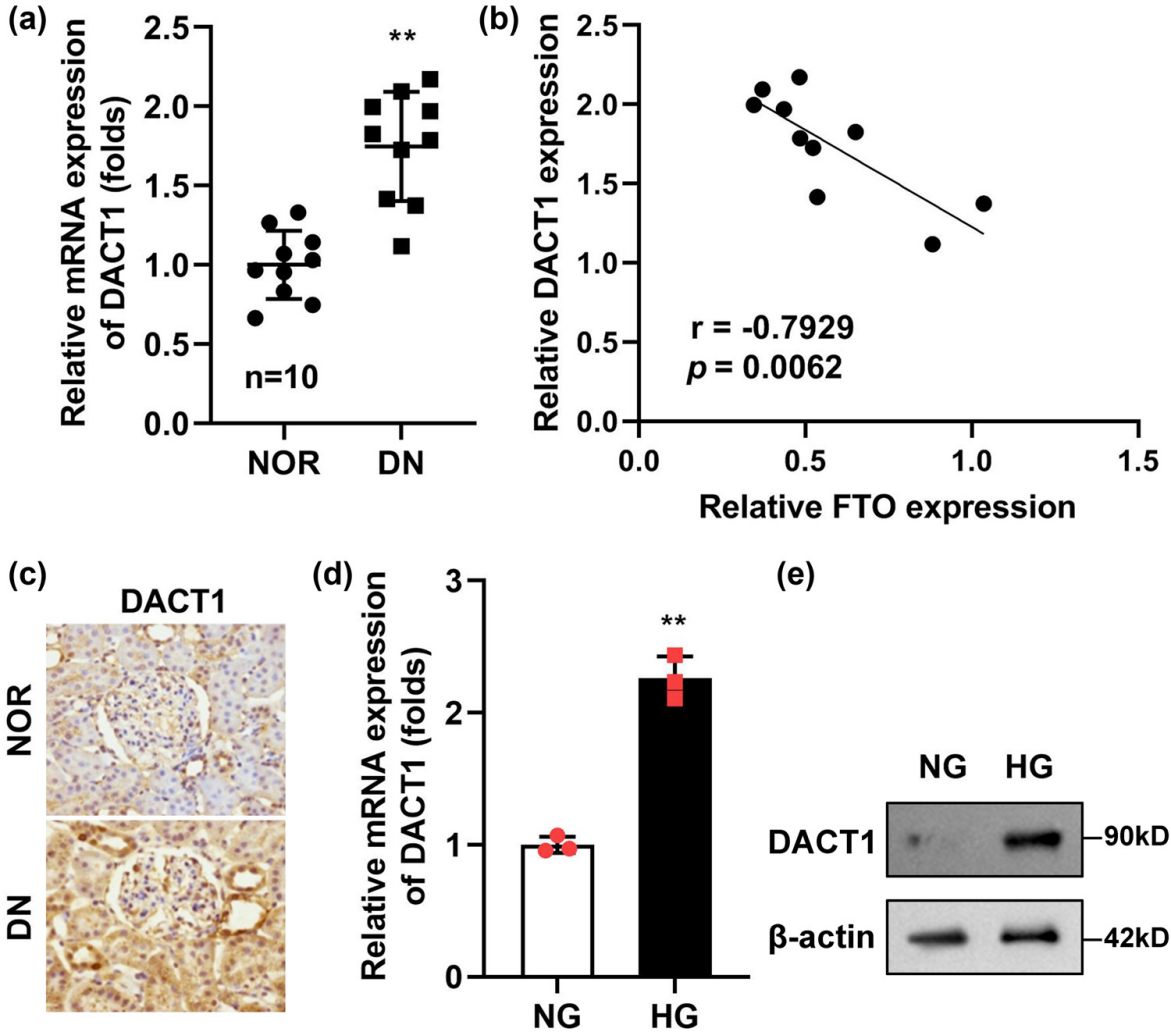
DACT1 was elevated in the HG-stimulated podocytes and renal tissues. (a) DACT1 was elevated in the renal tissues of DN patients (*n* = 10). (b) DACT1 was negatively correlated with FTO (*n* = 10). (c) The immunohistochemistry staining of DACT1 in the renal tissues of DN patients (*n* = 10). (d and e) The levels of DACT1 were determined with RT-qPCR and Western blot (*n* = 3). **P < 0.01.

As described in the introduction, DACT1 was significantly increased in diabetes. In the progression of osteosarcoma, the expression of DACT1 is regulated by the FTO-mediated m6A methylation modification. Therefore, we continued to analyze the correlation between DACT1 expression and FTO in DN. Compared with healthy renal tissues, DACT1 levels were markedly elevated in the renal tissues of DN patients (Figure 3a). Importantly, a negative correlation was observed between DACT1 and FTO expression in these tissues (Figure 3b), suggesting an inverse regulatory relationship between the two proteins. This finding was further supported by immunohistochemistry, which demonstrated high levels of DACT1 in the renal tissues of DN patients (Figure 3c). In the HG-stimulated podocytes, the mRNA (Figure 3d) and protein levels (Figure 3e) of DACT1 were prominently elevated.

### 3.4 Overexpressed FTO declined the m6A methylation modification of DACT1

**Figure 4:**
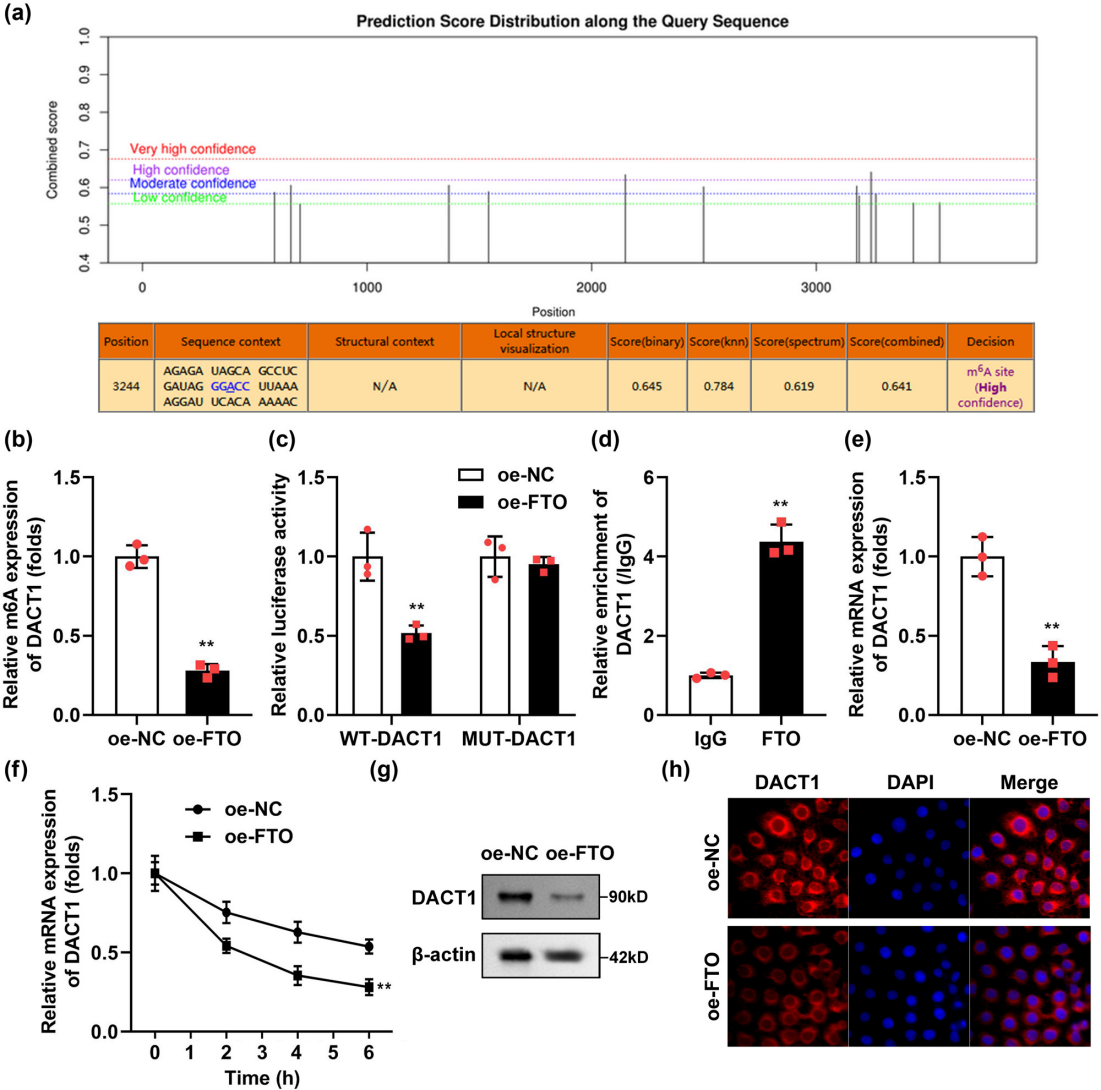
Overexpressed FTO declined the m6A methylation and mRNA levels of DACT1. (a) The m6A methylation sites of DACT1 were predicted using the SRAMP database. (b) After FTO overexpression, the m6A methylation levels of DACT1 were prominently declined. (c) The luciferase activity of WT-DACT1 was prominently depleted after FTO overexpression. (d) The RIP assay showed the FTO antibody specifically enriched the DACT1 mRNA in podocytes. (e and f) After FTO overexpression, the mRNA expression and stability of DACT1 were prominently declined. (g and h) Western blot and immunofluorescence of DACT1 (*n* = 3). **P < 0.01.

Given our finding that the expression of FTO and DACT1 in DN exhibits a negative correlation, we proceeded to investigate the m6A methylation modification mechanism of DACT1 by FTO, to clarify how FTO regulates the expression of DACT1. The SRAMP database (http://www.cuilab.cn/sramp) results showed that there were multiple methylation binding sites of DACT1 (Figure 4a). After FTO overexpression, the m6A methylation levels of DACT1 were significantly declined (Figure 4b). The luciferase activity of WT-DACT1 was significantly declined after FTO overexpression (Figure 4c). The RIP assay demonstrated that the FTO could bind to DACT1 mRNA (Figure 4d). The luciferase reporter and RIP assay results indicated that the interaction between FTO and DACT1 occurs at the m6A methylation modification site of DACT1. After FTO overexpression, the mRNA expression (Figure 4e) as well as stability (Figure 4f) of DACT1 were prominently declined. The protein levels of DACT1 were also declined after FTO overexpression (Figure 4g), the results of immunofluorescence showed the same results (Figure 4h).

### 3.5 Overexpressed DACT1 reversed the function of FTO in the HG-treated podocytes

**Figure 5:**
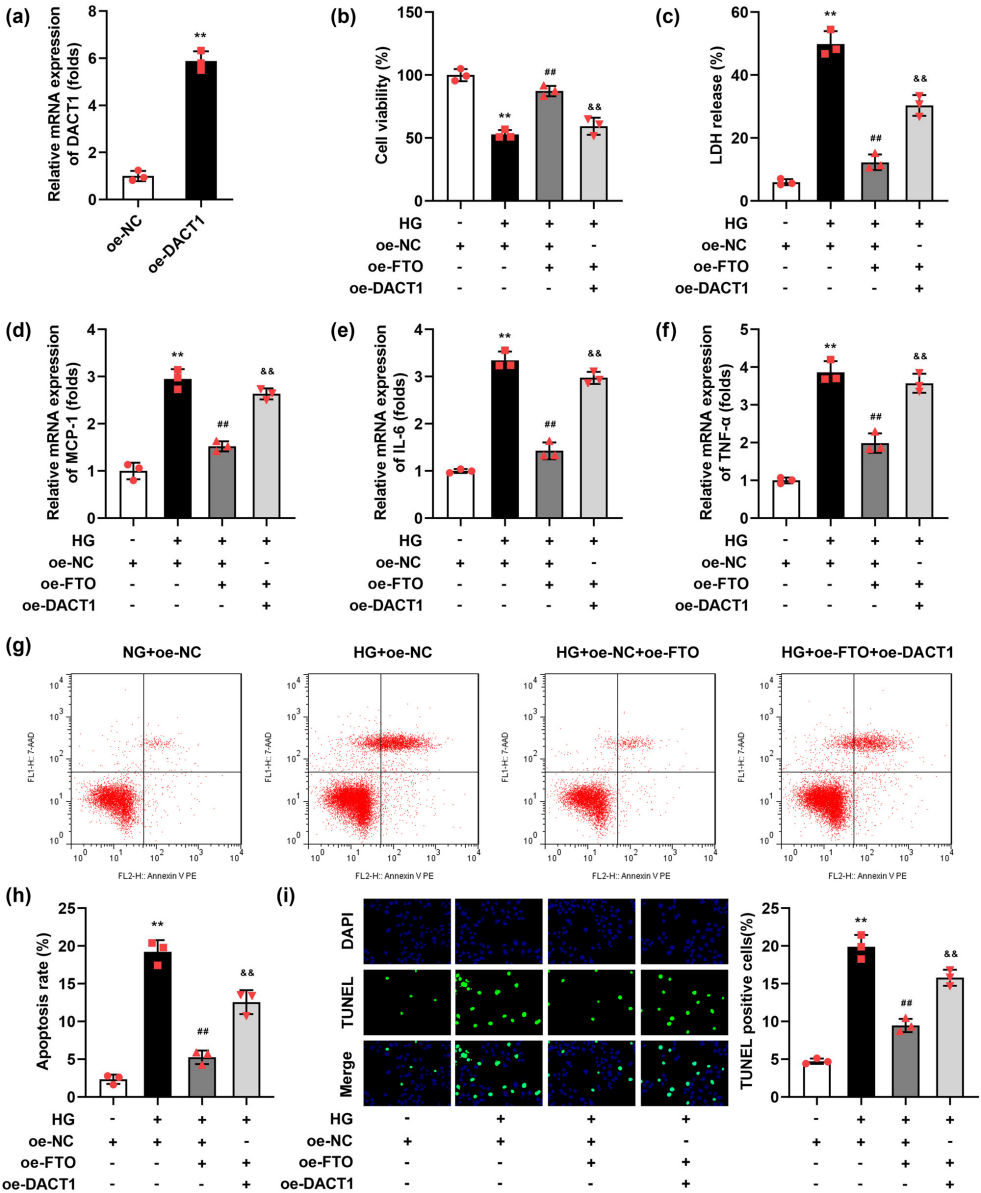
Overexpressed DACT1 neutralized the role of FTO in the HG-stimulated podocytes. (a) Validation of transfection efficiency of oe-DACT1. (b) The cell viability was detected by CCK-8 assay. (c) The LDH release was assessed with a kit. (d–f) The mRNA levels of MCP-1, IL-6, and TNF-α were tested with RT-qPCR. (g and h) Flow cytometry was performed to detect the cell apoptosis. (i) TUNEL staining was conducted to detect the cell death (*n* = 3). **P < 0.01 vs oe-NC group, ^##^ *P* < 0.01 vs HG + oe-NC group, ^&&^ *P* < 0.01 vs HG + oe-NC + oe-FTO group.

Having understood the regulatory role of FTO in the m6A methylation modification and expression of DACT1, we subsequently carried out rescue experiments to demonstrate whether, in the DN cell model, FTO participates in the regulation of cellular inflammation and apoptosis by downregulating the expression of DACT1. The DACT1 transfected podocytes showed a high mRNA expression of DACT1 (Figure 5a). In the HG stimulated and FTO transfected podocytes, after DACT1 transfection, the LDH release (Figure 5b), mRNA expression of MCP-1 (Figure 5c), IL-6 (Figure 5d), TNF-α (Figure 5e), apoptosis rate (Figure 5f and g), and TUNEL-positive cells (Figure 5h) were prominently elevated.

### 3.6 IGF2BP1 was the m6A methylation modification recognition reader of DACT1

**Figure 6:**
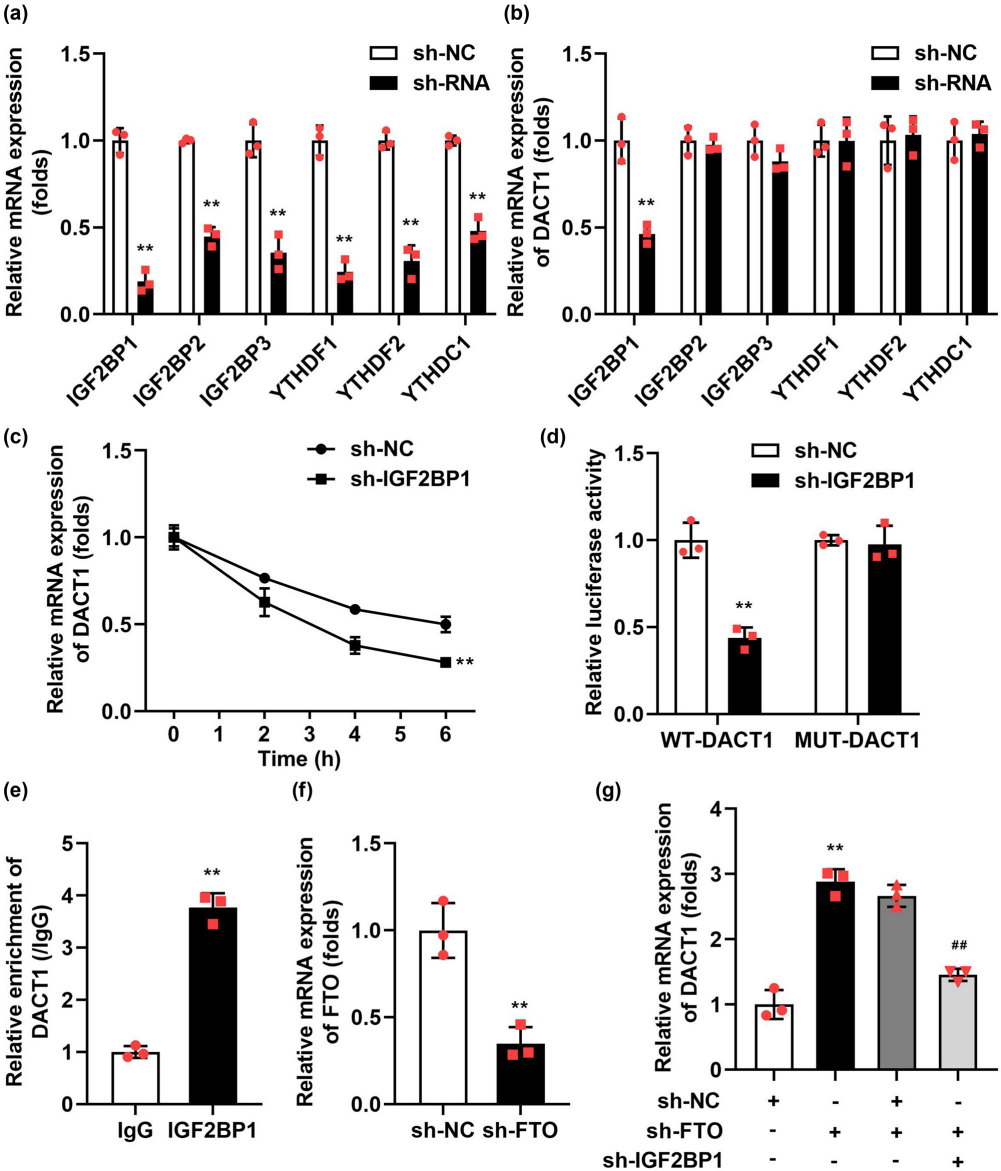
IGF2BP1-degraded DACT1 mRNAs in an m6A-dependent manner. (a) Validation of transfection efficiency of sh-RNAs. (b) After sh-IGF2BP1 transfection, the DACT1 levels were prominently declined. (c) Sh-IGF2BP1 also prominently depleted the mRNA stability of DACT1. (d) The luciferase activity of WT-DACT1 was prominently depleted after IGF2BP1 knockdown. (e) The RIP assay showed the IGF2BP1 antibody specifically enriched the DACT1 mRNA in podocytes. (f) Validation of transfection efficiency of sh-FTO. (g) After sh-FTO and sh-IGF2BP1 transfection, the DACT1 levels were assessed by RT-qPCR (*n* = 3). **P < 0.01.

The final regulation of gene expression by m6A methylation modification depends on the reader’s recognition of m6A methylation modification. Finally, we further explored which reader could recognize the m6A methylation-modified reader of DCT1. After sh-RNAs’ transfection, the expressions of m6A methylation modification readers (IGF2BP1, IGF2BP2, IGF2BP3, YTHDF1, YTHDF2 and YTHDC1) were prominently declined (Figure 6a). Additionally, IGF2BP1 knockdown decreased the DACT1 levels (Figure 6b). Besides, sh-IGF2BP1 prominently depleted the mRNA stability of DACT1 (Figure 6c). The luciferase activity of WT-DACT1 was prominently depleted after IGF2BP1 knockdown (Figure 6d). The RIP assay demonstrated that the IGF2BP1 could bind to DACT1 mRNA (Figure 6e). Furthermore, sh-FTO prominently declined the FTO levels in the podocytes (Figure 6f). Finally, we confirmed that IGF2BP1 knockdown reversed the effects of sh-FTO on the expression levels of DACT1 (Figure 6g).

## 4 Discussion

In this study, we demonstrated that FTO epigenetically inhibits DACT1 expression through an m6A-IGF2BP1-dependent mechanism. Moreover, we confirmed that FTO expression is significantly downregulated in podocytes stimulated by HG and in the kidneys of DN patients. FTO overexpression alleviated the injury of HG-stimulated podocytes.

Recent studies have increasingly highlighted the critical role of m6A methylation in RNA metabolism, influencing various cellular processes such as mRNA stability, splicing, and translation [23,24]. As the first identified m6A demethylase, FTO plays a pivotal role in removing m6A modifications from target RNAs, thereby regulating their expression and function [25]. FTO belongs to the AlkB family of non-heme iron(ii)-dependent and α-ketoglutarate-dependent dioxygenases, which are known to modulate various biological processes, including body mass and obesity development [26]. Previous research has shown that inhibition of FTO can prevent obesity and lead to growth retardation, underscoring its importance in metabolic regulation. Moreover, emerging evidence indicates that FTO exerts regulatory effects on mRNA expression in diverse pathological contexts. For instance, Niu et al. [27] demonstrated that FTO reduces the m6A methylation levels of BNIP3, accelerating its degradation and promoting breast cancer development. Shen et al. [28] found that FTO suppresses hypoxia/reoxygenation-induced apoptosis in myocardial cells by modulating the m6A methylation of Mhrt, suggesting its potential as a therapeutic target for heart failure. In DN progressions, Taira et al. [29] confirmed the dramatic relationship between rs56094641 in FTO and susceptibility to DN patients in Japan through genome-wide association studies. However, the specific mechanisms underlying FTO’s role in DN remain largely unexplored. In this research, we found that FTO was declined in the HG-stimulated podocytes and renal tissues of DN patients. FOT overexpression alleviated the injury, including LDH and inflammatory factors (MCP-1, IL-6, as well as TNF-α) release, induced by HG in the podocytes. These results indicated that increasing the FTO levels might be a promising therapeutic method for DN.

While numerous studies have demonstrated that FTO can promote the progression of obesity [30] and diabetes [31]. For the opposite phenomenon observed in this study, FTO expression was reduced in DN patients. We speculate that the downregulation of FTO observed in DN samples relative to normal (NOR) controls may be a consequence of chronic hyperglycemia and oxidative stress, which are hallmark features of diabetes. These conditions can lead to alterations in epigenetic modifiers as part of a compensatory response or due to impaired cellular homeostasis. While upregulation of FTO has been reported in some contexts related to diabetes, such as obesity, it is not universally observed across all diabetic complications. Therefore, the decrease in FTO expression in DN could reflect a specific aspect of this complication’s pathophysiology.

DACT1, recently identified as a tumor suppressor gene, has been demonstrated to be regulated by FTO through the m6A methylation modification [22]. Shi et al. [32] demonstrated that DACT1 overexpression depressed cell metastasis and accelerated cell death in cervical cancer. Similar antitumor effects of DACT1 have been reported in other malignancies, including breast cancer [33], lung cancer [34], gastric cancer [35], and so on. In the context of DN, the role of DACT1 has not been extensively studied. In our research, we found that DACT1 was overexpressed in the HG-stimulated podocytes and renal tissues of DN patients. Through the Double Luciferase Report and RIP experiment, we demonstrated the existence of interaction between FTO and DACT1. SRAMP database confirmed that multiple m6A methylation-modified sites existed on DACT1 mRNA. Meanwhile, we found that FTO overexpression depleted the m6A methylation levels of DACT1 and exacerbated the degradation of DACT1. Rescue experiments further illustrated that DACT1 overexpression reversed the role of FTO in HG-treated podocytes. All these findings suggested that FTO participated in the DN progression by modulating the m6A methylation levels of DACT1. As reported by Lv et al. [22], they also demonstrated that FTO decreased the mRNA expression and stability of DACT1 by regulating the m6A methylation levels in osteosarcoma. In this study, we have demonstrated for the first time that FTO regulates DACT1 modification in the context of DN, extending our understanding of the FTO/DACT1 signaling axis beyond its previously established role in tumors. In the future, we can continue to explore the role of this signaling axis in other diseases, to verify the universality of the FTO/DACT1 signaling axis and provide a solid theoretical basis for the development of clinical molecular targeted drugs.

The stability of target genes during m6A methylation modification is critically influenced by the recognition of m6A marks by “reader” proteins [36]. These readers play a pivotal role in regulating the expression of target genes, thereby participating in the progression of various diseases [37]. IGF2BP1, as a new family of m6A readers, has been confirmed to enhance the mRNA stability of target genes [38]. As an m6A reader, IGF2BP1 acts as an oncogenic factor in various cancer cells by stabilizing the expression of methylated mRNAs associated with oncogenic genes [39,40]. In addition, Huang et al. [41] demonstrated that IGF2BP1 promotes mRNA stability in an m6A-dependent manner, preferentially recognizing m6A methylation sites on mRNA. This selective recognition ultimately regulates the expression levels of related genes, underscoring the importance of IGF2BP1 in the post-transcriptional regulation of gene expression. In this study, through knockouting the expressions of readers, we found that IGF2BP1 knockdown declined the DACT1 expressions. The double luciferase report and RIP experiment confirmed the existence of interaction between IGF2BP1 and DACT1. Further experiments indicated that IGF2BP1 knockdown decreased the stability of DACT1 and neutralized the sh-FTO effects on DACT1 levels. Understanding the molecular mechanisms by which FTO regulates m6A methylation levels and interacts with IGF2BP1 provides a foundation for developing therapies that modulate these interactions rather than directly altering FTO expression. This approach could offer a safer alternative with reduced side effects.

However, there were still some limitations in this study. For RNA stability determination of DACT1, using only actinomycin D is not enough, and pulse tracking experiments are needed to further prove this. However, due to limited conditions, this will be the focus of our future research. On the other hand, the concern about the knockdown of DACT1 being potentially oncogenic is valid. However, it is important to note that the knockdown was performed in an *in vitro* model of DN, not in a cancer setting. The implications of DACT1 knockdown in DN need to be carefully evaluated in more specific models to ensure safety. Furthermore, any potential therapeutic strategy involving DACT1 would require extensive preclinical testing to assess both efficacy and safety. Exploring small molecules or RNA-based therapies that can selectively interfere with the DACT1–IGF2BP1 interaction might offer a safer alternative compared to direct knockdown.

To sum up, our study unveiled that low levels of FTO were a key factor for DN progression. Mechanistically, FTO overexpression inhibited the inflammatory reaction and cell death in the HG-stimulated podocytes by down-regulating the DACT1 levels. FTO-medicated m6A methylation modification of DACT1 was dependent on IGF2BP1.

## Supplementary Material

Supplementary Figure
