# Supplementary material for: FTO alleviated the diabetic nephropathy progression by regulating the N6-methyladenosine levels of DACT1
Source: Open Life Sci. 2025 May 5;20(1):20221049. doi: 10.1515/biol-2022-1049 (PMC12068186; doi:10.1515/biol-2022-1049)
Supplement: Supplementary Figure [file biol-2022-1049-sm.pdf]

Supplementary material

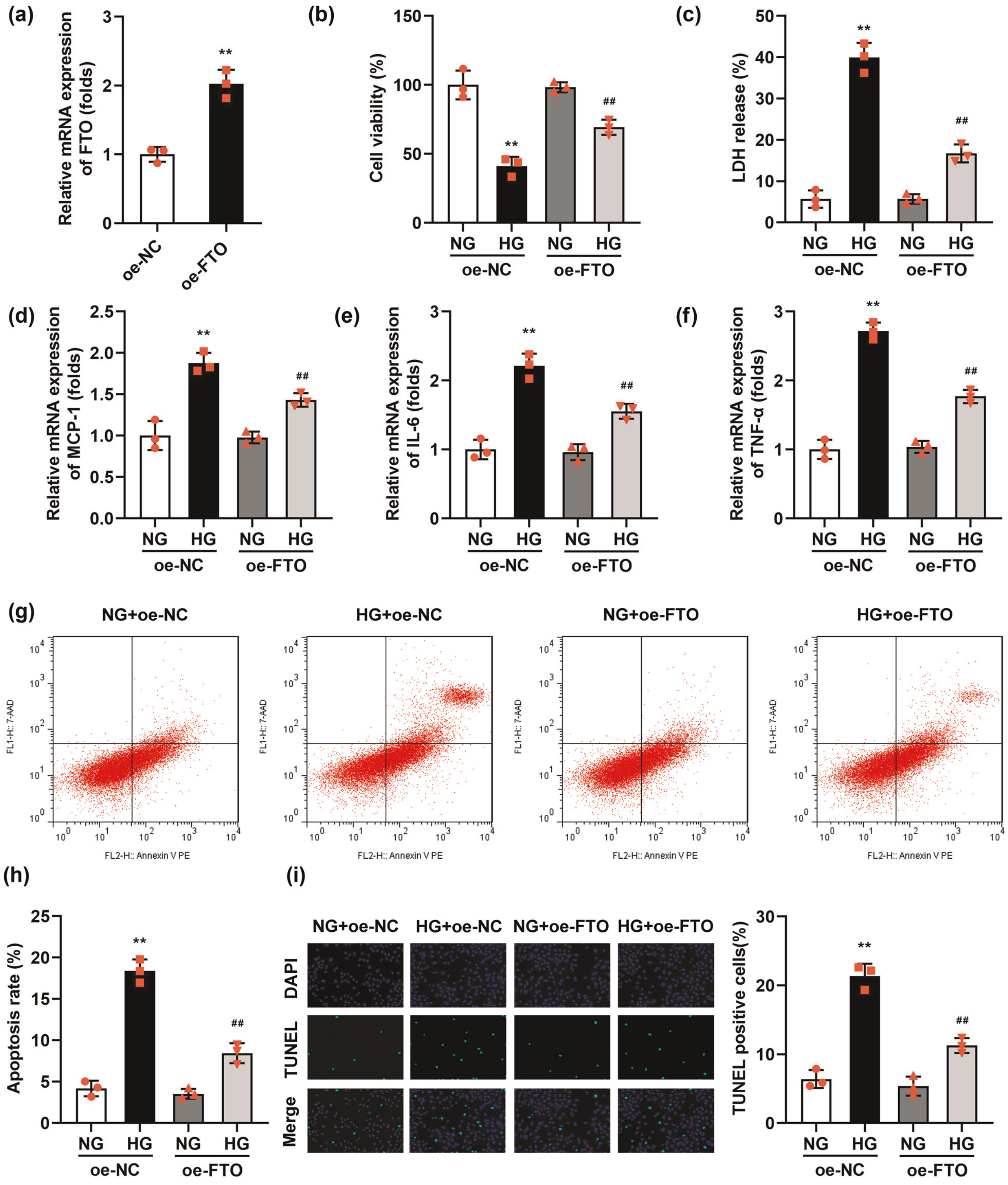

Figure S1: Overexpressed FTO relieved the injury of primary isolated podocytes induced by HG. (a) Validation of transfection efficiency of oe-FTO. (b) The cell viability was detected by CCK-8 assay. (c) The LDH release was assessed with kit. (d)–(f) The mRNA levels of MCP-1, IL-6 and TNF-α were tested with RT-qPCR. (g) and (h) Flow cytometry was performed to detect the cell apoptosis. (i) TUNEL staining was conducted to detect the cell death. (*n* = 3). ***P* < 0.01 VS NG + oe-NC group, ##*P* < 0.01 VS HG + oe-FTO.
